# Qualitative behavioral assessment of dogs with acute pain

**DOI:** 10.1371/journal.pone.0305925

**Published:** 2024-06-21

**Authors:** Francesca Zanusso, Barbara Contiero, Simona Normando, Flaviana Gottardo, Giulia Maria De Benedictis

**Affiliations:** 1 Department of Animal Medicine, Production and Health, University of Padova, Legnaro, Italy; 2 Department of Comparative Biomedicine and Food Science, University of Padova, Legnaro, Italy; Universidade do Porto Instituto de Biologia Molecular e Celular, PORTUGAL

## Abstract

Free Choice Profiling (FCP) methodology allows observers to qualitatively assess animal behavior using their own vocabulary. This study aims to investigate the ability of 3 different observer groups to recognize pain-related emotions in 20 dogs using FCP methodology, and to compare FCP data with the Glasgow Composite Pain Scale-Short Form (GCPS- SF) scores. The observer groups consisted of 10 dog owners, 10 veterinary students and 10 veterinarians. Ten healthy (“healthy”) dogs and 10 dogs showing clinical signs of pain (“pain”) were filmed, and the resulting 20 footages were shown to observers who were blind to the pain-related nature of the study. All observers described and scored animals’ emotional expression using FCP; then, students and veterinarians scored all dogs using GCPS- SF. FCP data were analyzed using Generalized Procrustes Analysis (GPA). Spearman correlation coefficient (ρ) was used to determine the correlation among observer groups’ FCP scores of the first two FCP dimensions (DIM1 and DIM2), and to compare GCPS-SF scores with FCP scores for the students and veterinarian observer groups. Each observer group reached a significant (p < 0.001) good consensus profile. “Healthy” dogs were mainly described as “quiet” and “lively”, while the majority of “pain” dogs were considered “in pain” and “suffering”. The correlation among FCP scores was high between owners’ DIM1 and students’ DIM1 (ρ = -0.86), owners’ DIM2 and students’ DIM2 (ρ = 0.72) and students’ DIM2 and vets’ DIM1 (ρ = 0.70). The correlation between GCPS-SF scores and FCP scores was high for students’ DIM2 (ρ = 0.77) and for veterinarians’ DIM1 (ρ = 0.92). Qualitative methods such as FCP could be used in association with semi-quantitative methods to evaluate the effect of pain on animal emotional expression. Observers’ cultural background and personal experience did not substantially affect qualitative behavioral assessment in dogs with acute somatic pain.

## Introduction

Free Choice Profiling (FCP) methodology was initially developed in food science [1–5] and later applied to qualitative behavioral assessment (QBA) in animals for the first time by Wemelsfelder et al. [6]. This approach has been successfully used in many animal species, including pigs [7, 8], cattle [9], horses [10–13], sheep [14], dairy buffalos [15, 16], giraffes [17], elephants [18] and dogs [19, 20]. QBA enables the assessment of the emotional status of the animals and does not rely on what an animal does, but on how it does what it does. The FCP approach allows observers to generate their own vocabulary to describe the emotional expression of observed animals, thereby enhancing active interpretation of animals rather than providing them with a fixed list of terms [7]. The FCP methodology has been applied to pain assessment in lambs [21, 22], but not to pain assessment in dogs.

Pain is not only a sensory but also an emotional experience [23]. Thus, physiological signs should not be relied upon as the sole indicators of pain [24, 25]. In contrast to humans, who can usually communicate their emotional states using verbal or written communication, animals lack verbal means of communication [26]. Thus, the ability of human observers to detect animal emotions is essential [27]. In dogs, the ability of humans to detect and distinguish facial expressions is likely enhanced by the effects of domestication and close relationships with humans [28]. Several multidimensional pain scales have been developed to assess pain in dogs, integrating pain-related behavior, emotional, and physiological changes [29–33]. Although pain scales are considered easy to apply in clinical practice, they have some limitations. Firstly, multidimensional pain scales consist of a fixed list of terms and do not allow observers to generate their own terms. Moreover, the reliability of scores depends on the experience and training of the assessors [33]. Additionally, only few pain scales have been validated in dogs. The short form of Glasgow Composite Pain Scale (GCPS-SF) is validated to assess acute somatic pain and defines an intervention point: if a dog was scored ≥6 points, analgesic treatment should be given [33–38]. GCPS-SF has been developed for use by veterinarians in clinical practice, while no tools to assess acute pain by owners have been developed yet [39].

The hypothesis of the present study was that observers would be able to assess emotional status of suffering dogs using FCP, notwithstanding their differences in experience and cultural background. The first aim of this study was to apply FCP to healthy and in pain dogs to test the inter-observer reliability of assessing pain-related emotional expression. The second aim of the study was to compare QBA assessment between three groups of observers with different personal experience and cultural background. The third aim was to investigate the correlation between QBA data and GCPS-SF scores assigned by observers. Thus, a qualitative method was compared to a semi-quantitative method to assess pain in dogs.

## Materials and methods

Ethical approval for this study was obtained from the Animal-welfare Body of the University of Padova (OPBA Authorization number 51/2023). Written informed consent was obtained from all participants and all dog owners.

### Animals

Animal subjects involved in this study were 20 client-owned dogs admitted to the Veterinary Teaching Hospital of the University of Padova. The dogs were 11 males and 9 females of various breeds, aged from 2 to 11 years and weighting from 4 kg to 45 kg. The clinical cases are provided in S1 Table. Dogs were assessed by two veterinarians, an orthopedic surgeon and an anesthesiologist. Based on a pain-oriented clinical examination (i.e. withdrawal response, lameness, muscle tension), they were classified as either “pain” (n = 10) or “healthy” (n = 10). “Pain” dogs showed signs of acute somatic pain. “Healthy” dogs were presented for vaccination. All dogs were housed individually in a cage for at least 2 hours before video recording.

### Videos

Twenty videos were created, each corresponding to a clinical case, involving two operators: a cameraman and a veterinarian (FZ), between July 30^th^, 2023 and August 24^th^, 2023. Each dog was video-recorded using an iPhone 6 video camera, in three different phases, each lasting 5 minutes. In the first phase, the dog was filmed in the cage without interacting with the operators. In the second phase, the veterinarian invited the dog to come out of the cage by calling its name, put a leash on it, and invited it to walk close to the cage (room dimension: 5m x 4m). In the third phase, the veterinarian (FZ) palpated the dog for approximately one minute, applying gentle pressure to the whole body. In all the “healthy” dogs, the palpation was performed in the same manner: in a cranio-caudal direction for the body and in a disto-proximal direction for the limbs. In the “pain” group, palpation was conducted on the body and limbs in the direction from the non-painful area to the wound or sore area. A footage of 2 minutes duration was created for each animal, with each phase approximately the same length (40 seconds). From this material, a video containing 20 footages, showing “healthy” and “pain” dogs in random order, was produced. Each footage was followed by a 1.5-minute blank frame, which was then followed by the next video footage. Thus, the total duration of the video was 70 minutes.

### Observers

Three observer groups of Italian native speakers (10 observers per group) were involved in this study: dog owners, veterinary medicine students, and veterinarians. Students were informed about the study via email, and the first 10 respondents were selected. Owners were recruited by the authors from friends, students, and their friends’ and families’ circles, with the first 10 respondents being selected. Veterinarians were recruited by the authors through email, and the first 10 respondents were selected. All participants were asked to complete a questionnaire to collect general information such as their age, gender, education, and personal experience with companion animals. The first observer group was composed by 2 males and 8 females dog owners, aged from 19 to 61 years. They all owned one dog and spent at least 4 hours per day with their dog. The level of schooling of the group ranged from high school to university education. The second observer group included 4^th^ and 5^th^ year students of the Master’s Degree Course in Veterinary Medicine at the University of Padova. It consisted of 3 males and 7 females, aged from 22 to 30 years. They all attended the anesthesia and the ethology courses, and had some clinical experience, and five of them were dog owners. The veterinarians’ group consisted of 2 males and 8 females who had graduated from the University of Padova (n = 8) or from another Italian university, for at least 2 years. One worked in the Veterinary Teaching Hospital of the University of Padova and nine in a private veterinary clinic. They were all companion animal veterinarians, with four mainly anesthesiologists, while others worked in internal medicine, surgery, and diagnostic imaging. Moreover, seven of them owned at least one dog.

### Experimental procedures

All participants were asked to view the video twice (session 1 and 2) following the FCP, adapted from Wemelsfelder et al. [7], as described in later paragraphs. The students and the veterinarians took part in a third session, where they used the Italian version of the Glasgow Composite Pain Scale-Short Form to assess the animals. The three groups were all given the same written information and instructed to follow the same assessment procedures. None of the observers was informed about the pain-related nature of the study, so none were focused on observing pain in the animals, and none had previous experience with FCP. They were informed that the study focused on the reliability of a methodology for assessing the behavioral expressions of dogs, but they did not receive any information about the dogs’ clinical condition or “healthy”-“pain” classification. All the materials provided were in the Italian language. To ensure independence of individual assessments within a group, silence was strictly maintained during sessions, and observers were told to refrain from any discussion regarding their descriptors or ratings throughout the entire study. The scoring was done in person by all the participants in one room.

#### Observer session 1

In the first session, the observers received the same bundle of sheets containing instructions and an empty paper where they should write down the adjectives. They all viewed the video using a lecture theatre screen together in the same room and wrote down the terms that they considered the best descriptors of the behavioral expression of the dog, during the 1.5-minute blank frame. For each new footage, observers were free to choose as many terms as they considered appropriate and to re-use terms or select new terms for each dog. At the end of the session, observers were asked to review the list of adjectives. In case of opposite meaning of two words, the term with the negative connotation should be removed from the list. Thus, each observer compiled a set of terms describing the expressive repertoire of the 20 dogs (personal descriptive list).

#### Observer session 2

In the second session, each observer was given a bundle of 20 sheets, each containing their own personal descriptive list of terms rearranged in alphabetical order. The second session took place 7 days after the first session. The written instructions explained how to use their personal terminologies as a quantitative measurement tool. Each term in the list was set next to a Visual Analogue Scale (VAS) where the extreme left end (0 mm) was considered the minimum and the extreme right end (100 mm) the maximum intensity of the selected adjective. The observers watched the video as described before. At the end of each footage, during the 1.5-minute blank frame, the observers scored the dog recorded in the video by marking a line for each term at an appropriate point between 0 and 100 mm.

#### Observer session 3

The students and the veterinarians were given a bundle of sheets containing instructions and the Italian translation of the Glasgow Composite Pain Scale-Short Form. Briefly, the scale consists of four sections (A, B, C, and D), each containing several possible items, arranged in ascending order of pain intensity [35]. The observers watched the video for the third time, and during the 1.5-minute blank frame, they chose the appropriate score for each dog.

### Statistical analysis

#### Generalized Procrustes analysis

The score on every term for a dog was determined by measuring the distance (in mm) between the left minimum point of the scale and the point where the observer’s mark crossed the line. These scores were entered into a data matrix, one for each individual observer, defined by the number of dogs (n = 20) and the number of terms used by a particular observer. This produced 30 data sets (one for each observer), each containing scores for the 20 dogs on the different observers’ terms. These 30 data sets were analyzed using Generalized Procrustes Analysis (GPA) and Principal Component Analysis (PCA), applied through specialized statistical code for Genstat written by Dr Tony E.A. Hunter at Biomathematics and Statistics Scotland, University of Edinburgh (Genstat 2016, VSN International, UK)”.

Briefly, GPA calculates a consensus profile between observer assessments through complex pattern matching. It represents observer matrices in virtual space as multi-dimensional configurations, with each configuration’s dimensions determined by the number of terms generated by a particular observer. GPA captures the similarity in scoring patterns between observers through iterative transformations (translation, rotation/reflection, and scaling) that maintain relative inter-sample relationships within each configuration. The level of consensus is determined by the percentage of variation between observer configurations explained by the consensus profile [7]. GPA is designed to find a consensus between a given set of matrices, regardless of how variable the data are. The name Procrustes derived from the Greek mythology: he was an innkeeper in Attica who managed to fit his guests into his one-size beds by cutting or stretching their legs as necessary and either tying them to the ironwork [40]. Thus, the danger exists that the attained consensus profile could be an artifact of the statistical technique rather than a significant feature of the data set. Therefore, the statistical significance of this consensus is evaluated against a mean randomized profile, obtained by re-running GPA with randomized observer data sets a hundred times. A one-tailed Student’s t-test (n = 100) is used to determine whether the true consensus differs significantly from the mean randomized profile; a probability of p < 0.001 is generally taken to indicate that the consensus profile was a meaningful feature of the data set rather than a statistical artifact. Using Principal Coordinate Analysis (PCO) of the Procrustes statistic for each pair of observers, the distance between transformed observer configurations and the ‘best-of-fit’ can be projected visually in an Observer plot. PCO estimates the center of distributions of observers together with a standard deviation and draws a 95% confidence region. Observers lying outside this region are potentially outliers; that is, they may differ from the other observers in their assessment of the samples. Through the second component of the software, the PCA, the number of dimensions of the consensus profile is reduced to one or more main dimensions explaining the majority of variation between the observed animals [6]. These dimensions are subsequently interpreted by correlating them to the original individual observer data matrices. This step of the analysis produces two-dimensional individual observer interpretative Word charts. In each chart, all terms of a particular observer are correlated with the principal axes of the consensus profile. These observer Word charts can be used for the interpretation of the main dimensions: the higher a term correlates with an axis, the more weight it has as descriptor for that axis. The calculation of the consensus profile takes place independently of the semantic information provided by the terminologies chosen by the observers. Semantic interpretation of this consensus profile takes place after its calculation. Thus, GPA preserves semantic information as part of the analysis of object-based data sets, independently from the experimenter’s interpretation of that information, making it possible to investigate whether observers apply their qualitative vocabulary in similar ways to characterize a group of dogs.

#### Inter-group FCP correlation

Intraclass Correlation Coefficient (ICC) and Spearman’s correlation coefficient (ρ) were used to determine the correlation among observer groups’ FCP data. The agreement among the adjectives used by the three observer groups was calculated using ICC. This coefficient provided a measure of how much the terms used were common between observer groups. The level of agreement among the scores of individual animals on the first two dimensions of the consensus profile was assessed using ρ. The strength of agreement for a value of ICC < 0.39 was interpreted as “poor”, 0.40–0.59 as “fair”, 0.60–0.74 as “good”, and 0.75–1.00 as “excellent” [41]. The correlation was considered: very high positive (negative) correlation if 0.90<ρ<1.00 (-0.90<ρ<-1.00), high positive (negative) correlation if 0.70<ρ<0.90 (-0.70<ρ<-0.90), moderate positive (negative) correlation, if 0.50<ρ<0.70 (-0.50<ρ<-0.70), low positive (negative) correlation if 0.30<ρ<0.50 (-0.30<ρ<-0.50) and negligible correlation if 0.00<ρ<0.30 (0.00<ρ<-0.30), according to Mukaka [42].

#### Statistical analysis of GCPS-SF scores

The agreement among all students and among all veterinarians on scores of the dogs was assessed using Intraclass Correlation Coefficient (ICC). The correlation between the two observer groups was calculated using Spearman’s correlation coefficient (ρ). Additionally, the sensitivity and the specificity of the Italian version of GCPS-SF were calculated considering the dogs’ classification in the two groups (“healthy” and “pain”) and the intervention point of 6/24 [34]. Thus, the sensitivity is the ratio between “GCPS-positive” dogs (score ≥6/24) and “pain” dogs, while the specificity is the ratio between “GCPS-negative” dogs (score <6/24) and “healthy” dogs.

#### Correlation between FCP and GCPS-SF scores

To make a descriptive comparison between GCPS-SF scores and FCP assessments, two modified Dog Plots (one for “healthy*”* dogs and the other for “pain” dogs) were made for each observer group. These plots represent "healthy" dogs and "pain" dogs, with labels assigned based on GCPS-SF scores. Dogs are categorized as "GCPS-negative" if they received a GCPS-SF score <6/24 and "GCPS-positive" if they scored ≥6/24, considering the GCPS-SF intervention point of 6/24. Additionally, the average GCPS-SF scores assigned to the dogs by the students and by the veterinarians were statistically compared with their behavioral assessment scores (individual animals’ GPA scores for the first two dimensions of each observer group) by Spearman’s correlation coefficient (ρ).

## Results

### Free choice profiling

The consensus profiles of the three observer groups explained a good percentage of variation among the observers (owners: 69.55%, veterinary students: 63.91%, and veterinarians: 62.47%; p < 0.001) and differed significantly from the mean of 100 randomized profiles (Table 1). The observer plots of the three groups show good consensus among the observers, with the majority of observers lying within the 95% confidence region: only 3 owners, 3 students, and 2 veterinarians were outliers (lying outside the 95% confidence region). Observer plots are provided in S1 Fig.

**Table 1:** Significance of the observer groups’ assessment of behavioral expression in dogs.

	Owners	Students	Veterinarians
**Consensus profile (%)**	69.55	63.91	62.47
**Mean ± SD (randomized profile)**	62.87 ± 0.18	56.58 ± 0.28	53.55 ± 0.58
**T**_**99**_	64***	57.8***	55.7***

*** p<0.001.

The 30 observers participating in this study generated a total of 170 terms to describe the dogs’ emotional expression (average: 17 terms per observer, range: 6–29). The most used adjectives were *dolorante* (“sore/in pain”), *tranquillo* (“quiet”) and *impaurito* (“fearful”) (S2 Table). The dog owners generated 123 terms (average: 21 terms per observer, range: 12–29), the veterinary students 79 terms (average: 16.6 terms per observer, range: 11–28) and the veterinarians 59 terms (average: 13.7 terms per observer, range: 6–25). Two main dimensions of the consensus profiles were identified for each observer group, explaining 25.3% and 14.5%, 23.0% and 16.3%, and 25.4% and 22.5% of the variation between animals for dog owners, veterinary students, and veterinarians, respectively. Table 2 lists the terms with the highest positive and negative correlation to each of these dimensions. On the basis of this table, in owners, DIM1 and DIM2 were defined as “*vivace* (lively)/*sconfortato* (discouraged)” and “*calmo* (calm)/*agitato* (restless)”; in students, DIM and DIM2 were characterized as “*preoccupato* (worried)/*attento* (attentive)” and “*tranquillo* (quiet) /*infastidito* (annoyed)”; in veterinarians, DIM1 and DIM2 were defined as “*tranquillo* (quiet)/*dolorante* (in pain)” and “*agitato* (restless)/*depresso* (depressed)”. As examples of these dimensions, the word charts of owner n.1 and student n.4 are shown in S2A and S2B Fig respectively.

**Table 2:** Terms showing the highest (*r*-value >0.5) positive and negative correlations with the dimensions of the consensus profile. Terms are listed in descending order of *r*-value.

	Dimensions (explaining %)	Negative correlation	Positive correlation
**Owners**	DIM 1 (25.3)	***Vivace*** (**lively**), *caparbio* (stubborn), *attivo* (active), *attento* (attentive), *ribelle* (rebel), *allegro* (cheerful), *curioso* (curious), *agitato* (restless), *irrequieto* (restless), *fuggitivo* (fleeting), *contento* (happy), *giocoso* (playful), *scattante* (agile), *giocherellone* (playful), *festoso* (merry), *intraprendente* (enterprising), *arzillo* (spry), *impaziente* (impatient), *frenetico* (frenetic), *sereno* (serene)	***Sconfortato*** (**discouraged**), *rassegnato* (resigned), *emarginato* (excluded), *intontito* (dazed), *distrutto* (exhausted), *afflitto* (afflicted), *dubbioso* (doubtful), *dolorante* (in pain/sore), *calmo* (calm), *provato* (exhausted), *distaccato* (aloof), *stanco* (tired), i*ncerto* (uncertain), *intimorito* (scared), *perplesso* (perplexed), d*isinteressato* (disinterested), *abbattuto* (depressed), *sofferente* (suffering), *affaticato* (tired), *disorientato* (disoriented), *confuso* (confused), *sconsolato* (disconsolate), p *passivo* (passive), *tranquillo* (quiet), *triste* (sad)
	DIM 2 (14.5)	***Calmo*** (**calm**), *timoroso* (timorous), *attento* (attentive), *tranquillo* (quiet)	***Agitato*** (**restless**), *insofferente* (impatient), *nervoso* (nervous), *scalmanato* (agitated), *sofferente* (suffering), *remissivo* (compliant), *ribelle* (rebel), *disorientato* (disoriented), *bisognoso* (needy), *esagitato* (overexcited), *irrequieto* (restless), *contento* (happy), *frenetico* (frenetic), *dolorante* (in pain/sore), *intimorito* (scared), *debole* (weak), *intontito* (dazed)
**Students**	DIM 1 (23.0)	***Preoccupato*** (**worried**), *intontito* (dazed), *timoroso* (timorous), *spaesato* (disoriented), *dolorante* (in pain/sore), *abbattuto* (depressed), *impaurito* (fearful), d*ubbioso* (doubtful), *inibito* (inhibited), *sofferente* (suffering), *docile* (docile)	***Attento*** (**attentive**), *ansioso* (anxious), *vispo* (lively), *agitato* (restless), *esagitato* (overexcited), *vigile* (alert), *incuriosito* (curious), *sicuro* (confident), c*ollaborativo* (collaborative), *irrequieto* (restless), *attivo* (active), *eccitato* (excited), *impaziente* (impatient), *reattivo* (responsive), *attivo* (active)
	DIM 2 (16.3)	***Tranquillo*** (**quiet**), *rilassato* (relaxed), *abbattuto* (depressed), s*ereno* (serene), *docile* (docile), c*ollaborativo* (collaborative), *spensierato* (carefree)	***Infastidito*** (**annoyed**), *agitato* (restless), *dolorante* (in pain/sore), *esagitato* (overexcited), *irrequieto* (restless), *ansioso* (anxious), *stressato* (stressed), *suscettibile* (sensitive), *nervoso* (nervous)
**Veterinarians**	DIM 1 (25.4)	***Tranquillo*** (**quiet**), *curioso* (curious), *sereno* (serene), *fiducioso* (confident)	***Dolorante*** (**in pain/sore**), *sofferente* (suffering), *claudicante* (lame), *zoppicante* (lame), *riluttante* (reluctant) *disorientato* (disoriented), *algico* (sore/in pain), *abbattuto* (depressed), *depresso* (depressed), *spaurito* (frightened), *infastidito* (annoyed), *nervoso* (nervous), *impaurito* (fearful)
	DIM 2 (22.5)	***Agitato*** *(****restless****)*, *eccitato* (excited), *nervoso* (nervous), *vivace* (lively), *attento* (attentive), *irrequieto* (restless), *ansimante* (panting), *attivo* (active), *ansioso* (anxious), *stressato* (stressed), *iperesagitato* (over-overexcited)	***Depresso*** (**depressed**), *impassibile* (non responding), *tranquillo* (quiet), *calmo* (calm), *sofferente* (suffering), *annoiato* (bored), *abbattuto* (depressed), *incerto* (uncertain)

DIM1: dimension 1, DIM2: dimension 2.

**Fig 1:**
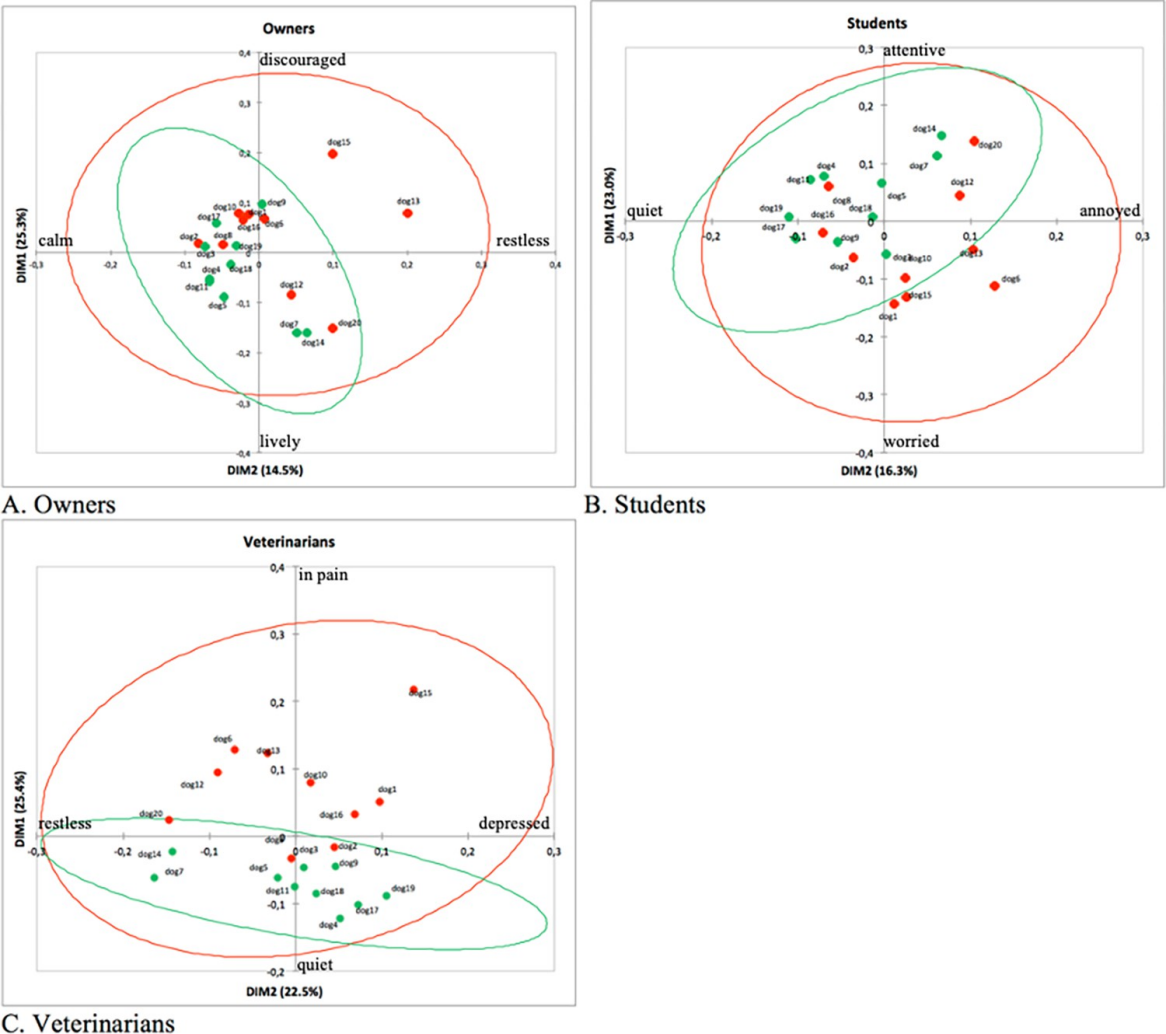
Dog plots. Distribution of the 20 dogs (“healthy”: green, “pain”: red) along dimensions 1 (DIM1) and dimension 2 (DIM2) of owners (A), students (B) and veterinarians (C).

Fig 1 shows the position of the individual dogs on the two main dimensions of each observer group. The dog groups are not evenly distributed over the dimensions in any of the three charts, suggesting that “pain” dogs were generally perceived to be in a state of reduced well-being compared to “healthy” dogs.

The degree of similarity among the terms used by pairs of observer groups was fair for owners vs students (ICC: 0.58); good for owners vs veterinarians (ICC: 0.67), and excellent for students vs veterinarians (ICC: 0.75). The correlation among the scores of individual animals was high between owners’ DIM1 and students’ DIM1 (ρ: -0.86), owners’ DIM2 and students’ DIM2 (ρ: 0.72) and students’ DIM2 and vets’ DIM1 (ρ: 0.70) (Table 3).

**Table 3:** Correlation between dog scores on the first two dimensions (DIM1 and DIM2) attributed by different observer groups (n = 20).

**Owners-students**		Owners DIM1	p-value	Owners DIM2	p-value
	Students DIM1	-0.86	< 0.001	0.04	0.872
	Students DIM2	-0.07	0.759	0.72	< 0.001
**Owners-veterinarians**		Owners DIM1		Owners DIM2	
	Veterinarians DIM1	0.47	0.037	0.60	0.005
	Veterinarians DIM2	0.61	0.005	-0.36	0.123
**Students-veterinarians**		Students DIM1		Students DIM2	
	Veterinarians DIM1	-0.52	0.018	0.70	< 0.001
	Veterinarians DIM2	-0.53	0.015	-0.65	0.003

DIM1: dimension 1, DIM2: dimension 2

### Glasgow Composite Pain Scale-Short Form

The intra-group agreement for students and veterinarians on dogs’ scores was excellent (ICC: students 0.92, veterinarians 0.93) and the inter-group agreement was very high (ρ: 0.93). On the other hand, out of the 10 dogs pre-classified as “pain”, only 6 were attributed with high GCPS-positive scores by students, and only 5 by veterinarians. The remaining “pain” dogs were given GCPS scores lower than 6 by both observer groups (Table 4).

**Table 4:** Dogs’ assessment using the Glasgow Composite Pain Scale (GCPS).

	Students		Veterinarians	
	GCPS-negative	GCPS-positive	GCPS-negative	GCPS-positive
**“healthy” dogs**	9	1	10	0
**“pain” dogs**	4	6	5	5

GCPS-negative: scores <6/24, GCPS-positive: scores ≥6/24. Mean GCPS score given by observers of each observer group was considered for each dog to assess it “GCPS-negative” or “GCPS-positive”.

### Correlation between FCP and GCPS-SF

**Fig 2:**
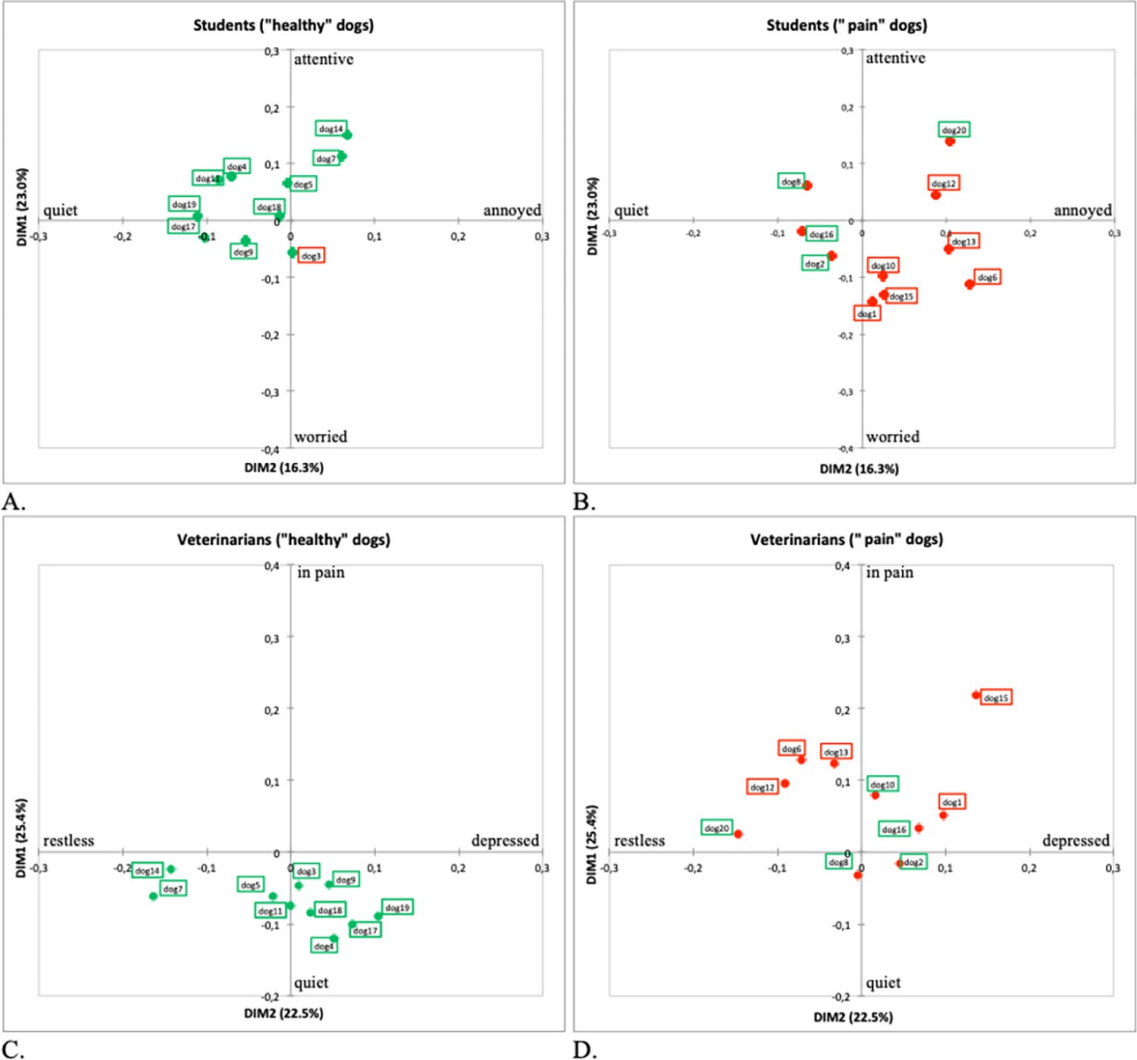
Dog plots (“healthy” and “pain”). Distribution of the 20 dogs (“healthy”: A, C and “pain”: B, D) along dimensions 1 (DIM1) and dimension 2 (DIM2) of students (A, B) and veterinarians (C, D). The color of the boxes around the dog numbers indicates the Glasgow Composite Pain Scale (GCPS) scores. Dogs in green boxes are “GCPS-negative” (scores <6/24) and those in red boxes are “GCPS-positive” (scores ≥6/24).

The average GCPS-SF scores for individual dogs were compared to their scores on the FCP dimensions (Fig 2). The most of “healthy” dogs were distributed toward the positive end of the first dimension for students, characterized mainly in terms such as *attento* (“attentive”), and these dogs had all been scored negatively by the students on the GCPS pain scale, except dog 3. For veterinarians, all the “healthy” dogs were distributed toward the negative end of the first dimension, characterized mainly by terms such as *tranquillo* (“quiet”), and all these dogs had been scored as GCPS-negative by the veterinarians. In contrast, “pain” dogs were distributed towards the positive end of the second dimension for students and the first dimension for veterinarians, characterized mainly by terms such as *infastidito* (“annoyed”) and *dolorante* (“in pain”). The different colored boxes for “pain” dogs in Fig 2B and 2D indicate that some of these dogs were given GCPS scores lower than 6 by students and veterinarians. The correlation between GCPS-SF scores and FCP scores was high for students’ DIM2 (ρ = 0.77) and for veterinarians’ DIM1 (ρ = 0.92). (Table 5).

**Table 5:** Correlation between Free Choice Profiling (FCP) dog scores and Glasgow Composite Pain Scale–Short Form (GCPS-SF) dog scores for students and veterinarians.

	Students GCPS-SF scores	p-value	Veterinarians GCPS-SF scores	p-value
FCP students DIM1 (worried/attentive)	-0.47	0.036	-0.56	0.010
FCP students DIM2 (quiet/annoyed)	0.77	<0.001	0.74	<0.001
FCP veterinarians DIM1 (quiet/in pain)	0.77	<0.001	0.92	<0.001
FCP veterinarians DIM2 (restless/depressed)	-0.19	0.431	-0.14	0.556

DIM1: dimension 1, DIM2: dimension 2, GCPS-SF: Glasgow Composite Pain Scale-Short Form, FCP: Free Choice Profiling

## Discussion

The results of this study support that QBA, using the Free Choice Profiling (FCP) methodology, can successfully applied to dogs [19, 20]. All observer groups achieved a good agreement using FCP, consistent with many other FCP studies [8, 11, 12, 15, 43–46]. Thus, observers from each group judged and quantified the emotional expression of the 20 dogs in a similar way. Consistent with other QBA studies involving different groups, observers with different background agreed on assessing the emotional expression of the animals [15, 47, 48]. Using QBA, all observers can converge on similar evaluations using this “whole-animal” methodology, which relies on the human ability to integrate details of behavior into judgments of animal “body language” [7]. It seems that no particular scientific knowledge is needed for QBA. In this study, the level of agreement among dog owners was even slightly higher than among students and veterinarians, supporting the idea that actual daily engagement with dogs is at least as relevant as the professional knowledge possessed by veterinarians and students in evaluating the emotional expression of dogs. It has been reported that a good inter-species communication between dogs and humans could have been developed during millennial domestication of dogs, making humans able to interpret this species probably better than others [48]. Moreover, the ability of owners to identify emotional states of their dogs based on animal behavior has been previously reported [49]. In this study, the vocabularies of the observer groups differed in width (i.e., the number of adjectives), but they shared many terms. The similarity between observer groups in assessing emotional expression of dogs is supported by the good degree of similarity among the terms used by the observer groups and by the strong correlation achieved between QBA dog scores on the dimensions attributed by the observer groups. Although the dimensions of the consensus profile were not identical among observer groups, terms such as *sofferente* (“suffering”) and *dolorante* (“in pain/sore”) were highly correlated with the first two dimensions in all observer groups. This suggests that all observers spontaneously assessed pain-related emotions, even though they were not informed about the aim of the study. Owners assessed dogs from *vivace* (“lively”) to *sconfortato* (“discouraged”), while for students and veterinarians, the emotional expression ranged from *tranquillo* (“quiet”) to *infastidito* (“annoyed”) or *dolorante* (“sore/in pain”). The owners’ dimensions were characterized by terms associated with discouragement, resignation, and tiredness, such as *sconfortato* (“discouraged”), *rassegnato* (“resigned”), *afflitto* (afflicted), *provato* (exhausted) and *stanco* (tired). For students, *dolorante* (“sore/in pain”) dogs were also *agitato* (“restless”), *preoccupato* (“worried”), *timoroso* (“timorous”), *inibito* (“inhibited”), *impaurito* (“fearful”). Students may have interpreted symptoms such as stress and anxiety as pain symptoms, which are often influenced by the context, for example by the so-called “white coat effect” [50–52]. Veterinarians preferred to define the pain in terms of physical alterations: a *dolorante* (“sore/in pain”) dog was also *zoppicante* (“lame”) or *riluttante* (“reluctant”). Thus, for veterinarians, medical words had more weight than for other groups as descriptors of the dimensions. The veterinarians’ first dimension was the clearest in terms of meaning, and consequently, the differentiation of the dogs between “healthy” and “pain” appeared the most evident among all observer groups. The “healthy” dogs were described as *vivace* (“lively”) or *attento* (“attentive”) or *tranquillo* (“quiet”), while the majority of the “pain” dogs were actually *scoraggiato* (“discouraged”) or *infastidito* (“annoyed”) or *dolorante* (“sore/in pain”).

Thus, the results of this study confirm that the qualitative behavior assessment methodology could be used to investigate pain in dogs, as reported in lambs [21, 22]. Pain is not only a sensory but also an emotional experience [23]. Thus, it is possible to differentiate “healthy” from “pain” dogs by focusing on animal emotional expression. In this study, pain-related emotions were perceived in a slightly different way by observer groups, since the dimensions of the consensus profile were similar but not the same among the observer groups, especially among veterinarians. The first dimension ranged from liveliness/activity to discouragement/worrying for owners and students, while for veterinarians, it ranged from quietness to pain. The second dimension ranged from calmness/quietness to restlessness in owners and students, while in veterinarians, it ranged from excitement to depression/suffering. It is worth noting that the first dimension of students was very similar in meaning to the first dimension of owners. Which terms are placed at which end of a dimension in GPA outcomes is arbitrary, so despite terms for students DIM1 in Table 2 being placed at opposite ends to those of owners’ DIM1, they describe a very similar emotional contrast. Thus, in our study, a slight difference between veterinarians and the other observer groups was observed. However, we cannot exclude the possibility that the difference between veterinarians and the other observer groups might be attributed to the terms used by veterinarians who did not fully follow the instruction of describing the emotional expression of dogs and instead used physical terms such as *zoppicante* ("lame"). While we could have removed these terms from the analysis, we believe that such removal could be perceived as a manipulation of the data. It is interesting to note that only veterinarians used physical terms, deviating from the instruction to describe the emotional expression of dogs. Veterinarians were understandably motivated to use professionally meaningful terms such as “lame”; however, inserting scores for a physical term may have altered the analysis of scores for emotional terms, in comparison to the other observer groups. For veterinarians, more pain-related terms were highly correlated with the first dimension, suggesting that the veterinarians’ experience in observing pain in dogs may have emphasized their ability to identify suffering dogs.

In this study, we compared FCP results with scores from a validated pain scale. It is important to note that QBA cannot be utilized to identify pain in the same manner as dedicated pain scales. When observers were tasked with assessing the emotional expression of dogs, their focus was not on pain assessment, and they were unaware of the pain-related nature of the study. However, an agreement between the results from a qualitative method with a validated semi-quantitative method could be useful to confirm the validity of the qualitative method. In our study, the use of this pain scale resulted in a higher level of agreement compared to the FCP assessment. Indeed, GCPS-SF was specifically developed to assess pain, while FCP is focused on the emotional status of the animals. In this study, 4^th^ and 5^th^ year veterinary students had enough knowledge to use the GCPS-SF as veterinarians did. Barletta et al. [34]. found that 1^st^ and 2^nd^ year students assessed pain in dogs differently from experienced anesthesiologists. Since scientific knowledge is needed to use pain scale, dog owners did not use GCPS-SF in this study. Both using FCP and GCPS-SF, the “healthy” dogs were identified more easily than the “pain” dogs. Some of the “pain” dogs were described as not very *dolorante* (“sore/in pain”) using FCP. These dogs were also given a GCPS score <6/24 by students and veterinarians, which indicates that the observers thought that these dogs were not suffering a lot and were not needing an analgesic treatment [34]. It is interesting to note that, whether using FCP or the pain scale, these “pain” dogs were categorized as only slightly sore, indicating a mild level of pain. The lack of all possible pain levels might represent a limitation of this study. In fact, dogs being in severe pain were not recruited in this clinical study, for ethical and clinical reasons: a very sore dog has to be treated as soon as possible and could not be filmed. However, the excellent correlation found in this study between the qualitative assessment (FCP) and the semi-quantitative method (GCPS-SF) suggests that FCP could potentially add valuable information to help the interpretation of semi- quantitative data. Although FCP alone may not be suitable as a standardized clinical tool, consideration should be given to integrating the QBA approach into pain management for companion animals. While not investigated in this study, a standardized QBA term list could potentially be useful for pain assessment in dogs. A recent study in shelter dogs proposed a promising fixed term list for welfare assessment [53].

The percentages explaining the two main dimensions were relatively low compared to many FCP studies [6, 7, 8–12, 19, 44–46]. This might be related to the complexity of visual information provided in videos in this study. To accommodate the use of the Glasgow Composite Pain Scale, dogs were filmed in three different situations, leading to a complex emotional status to describe. In this study, observers were asked to use terms in their mother tongue, because the FCP assessment has to give the observers complete freedom in generating terms [6]. Clearly, even though observers could speak another language very well, this freedom is actually possible only using their mother tongue. The Italian language has already been successfully used in a recent FCP study in which observers reached a high consensus profile assessing the emotional status of elephants, both using Italian terms both translating them into English [18].

## Conclusions

The results of the present QBA application in dogs confirm the reliability of this methodology in assessing emotional expression in canine species, adding an important contribution to the literature supporting QBA use in animals. Additionally, this FCP application to suffering animals demonstrates that QBA could be used to investigate the effect of pain on emotional expression. Differences in experience and cultural background do not appear to substantially affect the interpretation of the emotions expressed by suffering dogs. In this study, the excellent correlation reached by experienced observers between QBA assessment and the semi-quantitative pain assessment (GCPS-SF) suggests that FCP could be applied to pain assessment, opening the way to further QBA applications in suffering animals. Further studies are needed to investigate how cultural backgrounds could influence the assessment of pain-related emotions.

## Supporting information

S1 Fig Observer plots of owners (A), students (B) and veterinarians (C). The axes reflect Principal Coordinate Analysis (PCO) scaling values for relative observer distance, with numbers indicating individual observers. The dotted ellipse depicts the 95% confidence region for the normal population of observers.
(TIF)

S2 Fig Observer word charts. Shown as examples are word charts of owner 1 (A) and student 4 (B). The axes of this word chart show the first two main dimensions of the Generalized Procrustes Analysis (GPA) and indicate which of each particular observer terms best correlate with those dimensions.
(TIF)

S1 Table Description of clinical cases. Dogs are numbered according to the order in which they were watched by observers.
(DOCX)

S2 Table List of all terms used by observers. The second column indicates the total number of observers who used each adjective. The third, the fourth, and the fifth columns indicate how many owners, students and veterinarians, respectively, used each term.
(DOCX)
