# Supplementary figures and images for: Qualitative behavioral assessment of dogs with acute pain
Source: PLoS One. 2024 Jun 21;19(6):e0305925. doi: 10.1371/journal.pone.0305925 (PMC11192414; doi:10.1371/journal.pone.0305925)

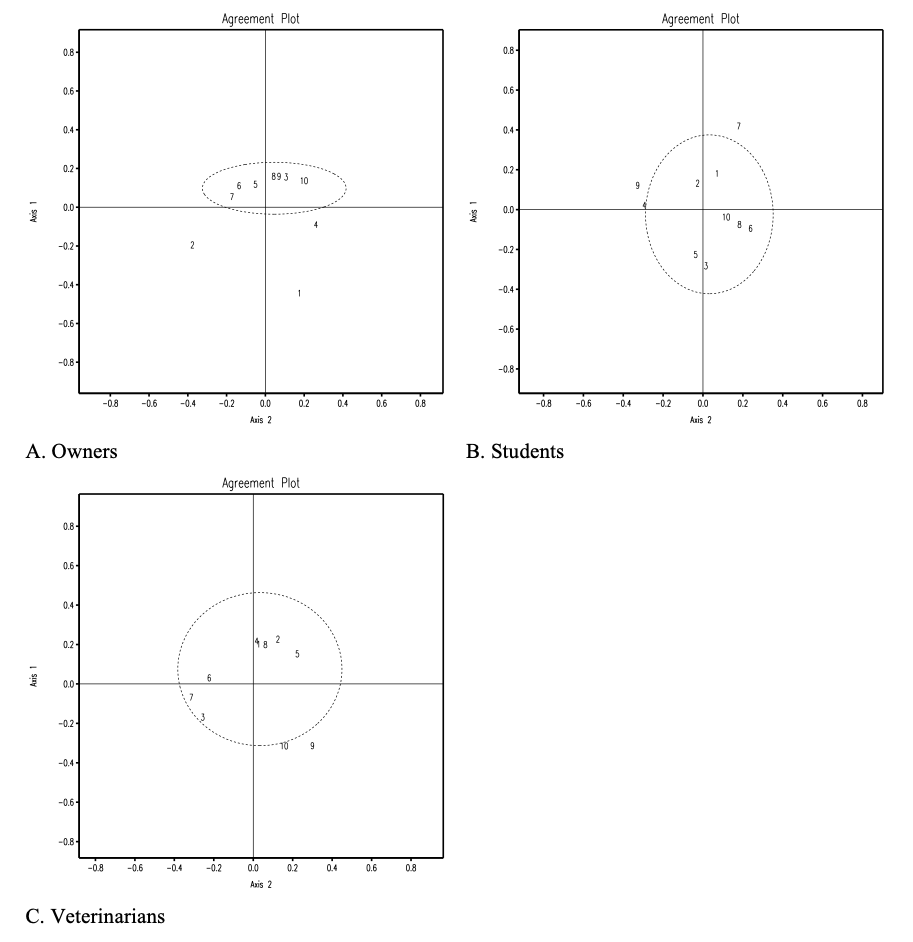

Supplement: S1 Fig — Observer plots of owners (A), students (B) and veterinarians (C). The axes reflect Principal Coordinate Analysis (PCO) scaling values for relative observer distance, with numbers indicating individual observers. The dotted ellipse depicts the 95% confidence region for the normal population of observers. (TIF) [file pone.0305925.s001.tif]

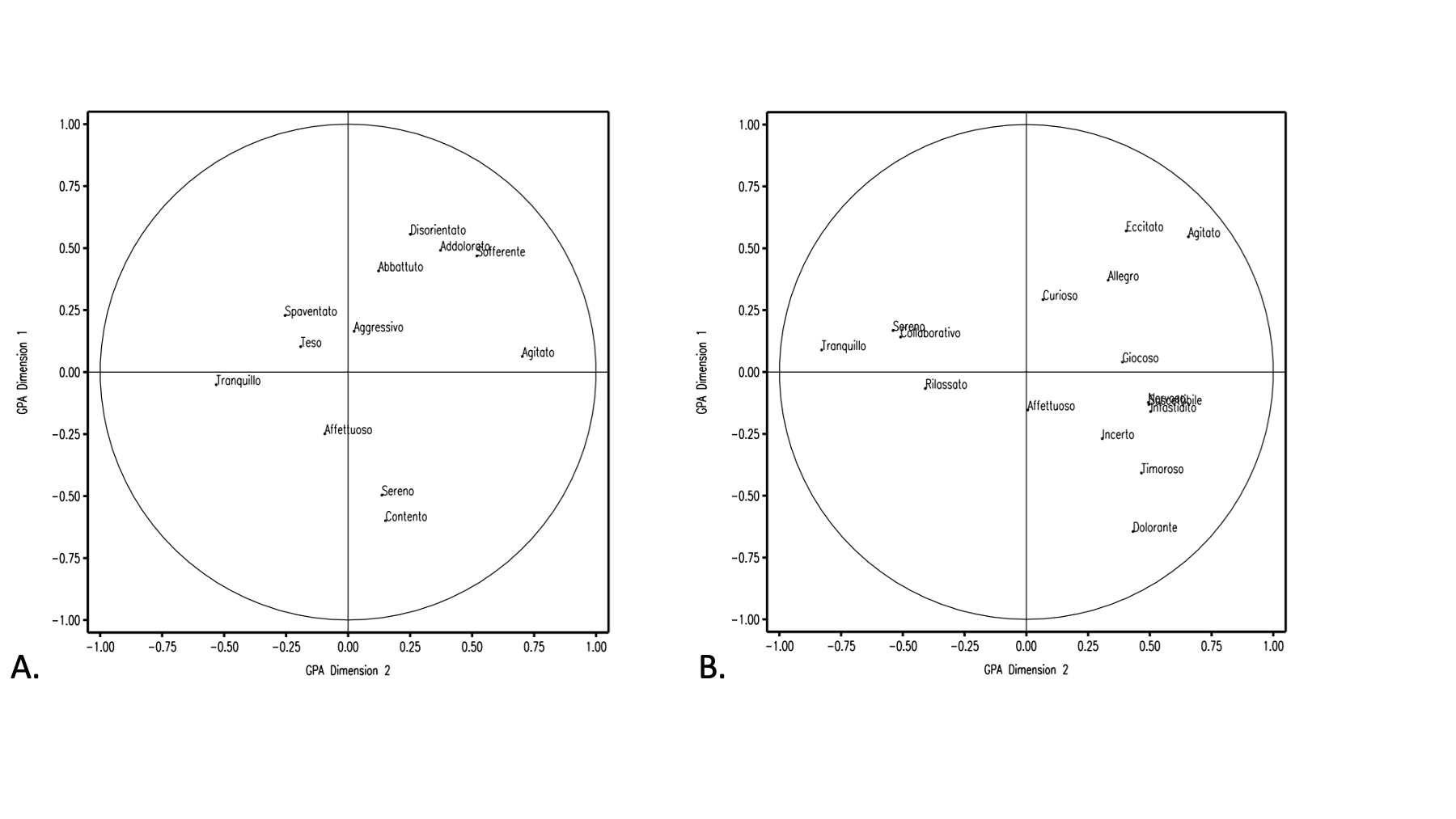

Supplement: S2 Fig — Shown as examples are word charts of owner 1 (A) and student 4 (B). The axes of this word chart show the first two main dimensions of the Generalized Procrustes Analysis (GPA) and indicate which of each particular observer terms best correlate with those dimensions. (TIF) [file pone.0305925.s002.tif]
