# Supplementary material for: Qualitative behavioral assessment of dogs with acute pain
Source: PLoS One. 2024 Jun 21;19(6):e0305925. doi: 10.1371/journal.pone.0305925 (PMC11192414; doi:10.1371/journal.pone.0305925)
Supplement: S1 Table — Dogs are numbered according to the order in which they were watched by observers. (DOCX) [file pone.0305925.s003.docx]

| **Clinical case** | **Age (years)** | **Weight (kg)**  **(kg)** | **Sex** | **“Helthy” (H) or “pain” (P)** | **Painful area** |
| --- | --- | --- | --- | --- | --- |
| **1** | 3 | 40 | M | P | left knee |
| **2** | 4 | 17 | F | P | right hip joint |
| **3** | 2 | 32 | M | H |  |
| **4** | 11 | 7 | M | H |  |
| **5** | 10 | 6 | F | H |  |
| **6** | 10 | 11 | F | P | right arm |
| **7** | 4 | 35 | M | H |  |
| **8** | 2 | 3 | M | P | left arm |
| **9** | 8 | 17 | F | H |  |
| **10** | 2 | 3 | F | P | right knee |
| **11** | 3 | 8 | M | H |  |
| **12** | 2 | 3 | M | P | left hip joint |
| **13** | 5 | 29 | M | P | left hock |
| **14** | 4 | 6 | M | H |  |
| **15** | 2 | 24 | F | P | right arm and hind limbs |
| **16** | 2 | 4 | M | P | left hind limb, thorax and left ear |
| **17** | 7 | 25 | M | H |  |
| **18** | 9 | 16 | F | H |  |
| **19** | 10 | 45 | F | H |  |
| **20** | 10 | 9 | F | P | left knee |
