# Supplementary material for: Qualitative behavioral assessment of dogs with acute pain
Source: PLoS One. 2024 Jun 21;19(6):e0305925. doi: 10.1371/journal.pone.0305925 (PMC11192414; doi:10.1371/journal.pone.0305925)
Supplement: S2 Table — The second column indicates the total number of observers who used each adjective. The third, the fourth, and the fifth columns indicate how many owners, students and veterinarians, respectively, used each term. (DOCX) [file pone.0305925.s004.docx]

| **Term** | **Observers** | **Owners** | **Students** | **Veterinarians** |
| --- | --- | --- | --- | --- |
| *Dolorante* (sore/in pain) | **21** | 6 | 7 | 8 |
| *Tranquillo* (quiet) | **20** | 8 | 5 | 7 |
| *Impaurito* (fearful) | **17** | 6 | 6 | 5 |
| *Agitato (restless)* | **16** | 5 | 4 | 7 |
| *Attento* (attentive) | **16** | 4 | 7 | 5 |
| *Abbattuto* (depressed) | **13** | 2 | 5 | 6 |
| *Nervoso* (nervous) | **12** | 2 | 3 | 7 |
| *Timoroso* (timorous) | **11** | 2 | 6 | 3 |
| *Curioso* (curious) | **11** | 2 | 5 | 4 |
| *Sofferente* (suffering) | **10** | 6 | 0 | 4 |
| *Disorientato* (disoriented) | **10** | 3 | 3 | 4 |
| *Contento* (happy) | **9** | 5 | 2 | 2 |
| *Infastidito* (annoyed) | **9** | 1 | 6 | 2 |
| *Vivace* (lively) | **8** | 4 | 0 | 4 |
| *Rilassato* (relaxed) | **8** | 3 | 3 | 2 |
| *Ansioso* (anxious) | **8** | 1 | 5 | 2 |
| *Irrequieto* (restless) | **7** | 4 | 2 | 1 |
| *Diffidente* (wary) | **7** | 3 | 4 | 0 |
| *Spaesato* (disoriented) | **7** | 3 | 3 | 1 |
| *Vigile* (alert) | **6** | 3 | 2 | 1 |
| *Affettuoso* (affectionate) | **6** | 2 | 3 | 1 |
| *Sereno* (serene) | **6** | 2 | 1 | 3 |
| *Indifferente* (unconcerned) | **6** | 2 | 0 | 4 |
| S*paventato* (scared) | **6** | 2 | 0 | 4 |
| *Collaborativo* (collaborative) | **6** | 1 | 5 | 0 |
| *Incerto* (uncertain) | **6** | 1 | 2 | 3 |
| *Triste* (sad) | **5** | 5 | 0 | 0 |
| *Incuriosito* (curious) | **5** | 2 | 3 | 0 |
| *Stressato* (stressed) | **5** | 1 | 2 | 2 |
| *Riluttante* (reluctant) | **5** | 0 | 2 | 3 |
| *Rassegnato* (resigned),  *stanco* (tired) | **4** | 3 | 1 | 0 |
| *Calmo* (calm) | **4** | 3 | 0 | 1 |
| *Allegro* (cheerful) | **4** | 2 | 2 | 0 |
| *Giocoso* (playful) | **4** | 1 | 3 | 0 |
| *Annoiato* (bored), *felice* (happy)*, guardingo* (cautious), *preoccupato* (worried) | **4** | 1 | 2 | 1 |
| *Attivo* (active) | **4** | 1 | 1 | 2 |
| *Depresso* (depressed), *sedato* (sedated) | **4** | 1 | 0 | 3 |
| *Eccitato* (excited) | **4** | 0 | 3 | 1 |
| *Aggressivo* (aggressive), *insofferente* (impatient), *ribelle* (rebel) | **3** | 3 | 0 | 0 |
| *Debole* (weak), *intontito* (dazed), *timido* (shy) | **3** | 2 | 1 | 0 |
| *Affaticato* (tired), *afflitto* (afflicted), *arzillo* (spry), *assetato* (thirsty), *confuso* (confused), *coraggioso* (brave), *giocherellone* (playful), *immobile* (still), *manipolabile* (able to be manipulated/submissive), *sveglio* (awake) | **2** | 2 | 0 | 0 |
| *Docile* (docile), *socievole* (sociable) | **3** | 1 | 2 | 0 |
| *Dubbioso* (doubtful), *esagitato* (overexcited), *sottomesso* (submissive) | **3** | 1 | 1 | 1 |
| *Rigido* (rigid), *zoppicante* (lame) | **3** | 0 | 1 | 2 |
| *Disinteressato* (disinterested), *frenetico* (frenetic), *impaziente* (impatient), *indispettito* (upset), *inibito* (inhibited), *paziente* (patient), *scattante* (agile), *sospettoso* (suspicious) | **2** | 1 | 1 | 0 |
| *Passivo* (passive) | **2** | 1 | 0 | 1 |
| *Determinato* (determinated), *impotente* (powerless), *insicuro* (insecure), *sicuro* (confident) | **2** | 0 | 2 | 0 |
| *Reattivo* (responsive), *stranito* (dazed) | **2** | 0 | 1 | 1 |
| *Atassico* (ataxic), *titubante* (undecided) | **2** | 0 | 0 | 2 |
| *Abbandonato* (neglected), *acccaldato* (hot)*,* *addolorato* (grieved), *affamato* (hungry), *assonnato* (sleepy), *azzoppato* (lame), *bisognoso* (needy), *caparbio* (stubborn), *cauto* (cautious), *composto* (composed), *comunicativo* (communicative), *dipendente* (dependent), *disobbediente* (disobedient), *disperato* (desperate), *disponibile* (helpful), *distaccato* (allof), *distrutto* (exhausted), *emarginato* (excluded), *fermo* (still), *festoso* (merry), *fuggitivo* (fleeting), *impuntato* (balked), *in allerta* (alert), *intimorito* (scared), *intorpidito* (numb), *intraprendente* (enterprising), *morente* (dying), o*ppositivo* (opponent), *pauroso* (fearful), *perplesso* (perplexed), *perso* (puzzled), *pimpante* (lively), *provato* (exhausted), *rabbioso* (furious), *remissivo* (compliant), *restio* (reluctant), *rimbambito* (doddering), *scalmanato* (agitated), *sconfortato* (discouraged), *sconsolato* (disconsolate), *seccato* (annoyed), *serio* (serious), *sicuro di sé* (self-confident), *spazientito* (on edge), *sperduto* (lost), *stordito* (dazed), *supplicante* (begging), *tenebroso* (mysterious), *teso* (tense), *testardo* (stubborn), *tontolone* (stupid), *tremante* (shaking), | **1** | 1 | 0 | 0 |
| A*ffranto* (disheartened), *amichevole* (friendly), *apatico* (apathetic), *assopito* (drowsy), *demoralizzato* (demoralized), *distratto* (distracted), *emozionato* (moved), *in forze* (strong),*incontenibile* (uncontainable), *insensibile* (insensitive), *interessato* (interested), *interrogativo* (questioning), *mogio* (dejected), *presente* (present), *schivo* (reserved), *scodinzolante* (wagging its tail), *spensierato* (carefree), *stufo* (sick and tired), *suscettibile* (sensitive), *vispo* (lively) | **1** | 0 | 1 | 0 |
| *Algico* (sore/in pain), *ansimante* (panting), *claudicante* (lame), *dispnoico* (dispnoic), *espansivo* (expansive), *fiducioso* (confident), *indeciso* (indecisive), *inquieto* (restless), *iperesagitato* (over-overexcited), *iperventilante* (hyperventilating), *positivo* (positive), *rallentato* (slow), *spaurito* (frightened), *stoico* (stoic) | **1** | 0 | 0 | 1 |
